# Self-Assembled Three-Dimensional Au Films as Highly Reproducible and “Hotspots”-Rich Substrates for Multiplex SERS Detection

**DOI:** 10.1021/acs.analchem.5c01716

**Published:** 2025-08-14

**Authors:** Rafael Villamil Carreón, José Juan. Gervacio-Arciniega, Ma. Estela Calixto, Siva Kumar Krishnan

**Affiliations:** ∥ Facultad de Ciencias Físico Matemáticas, Benemérita Universidad Autónoma de Puebla, Av. San Claudio y Blvd. 18 Sur, Col. San Manuel, Ciudad Universitaria, Puebla ,Pue. 72570, México; § SECIHTI- Facultad de Ciencias Físico Matemáticas, Benemérita Universidad Autónoma de Puebla, Av. San Claudio y Blvd. 18 Sur, Col. San Manuel, Ciudad Universitaria, Puebla, Pue. 72570, México; † Instituto de Física “Ing. Luis Rivera Terrazas”, 3972 Benemérita Universidad Autónoma de Puebla, Av. San Claudio y Blvd. 18 Sur, Col. San Manuel, Ciudad Universitaria, Puebla,Pue. 72570, México; # SECIHTI-Instituto de Física “Ing. Luis Rivera Terrazas”, 3972 Benemérita Universidad Autónoma de Puebla, Av. San Claudio y Blvd. 18 Sur, Col. San Manuel, Ciudad Universitaria, Puebla,Pue. 72570,México

## Abstract

Three-dimensional (3D) plasmonic metal nanostructures show great promise for surface-enhanced Raman scattering (SERS) detection of analyte molecules. However, obtaining uniform “hotspots” is still a paramount challenge. Herein, we report a low-cost strategy for the scalable preparation of 3D Au films with a high density of “hotspots” using a deep eutectic solvent (DES)-mediated interfacial self-assembly of thermally evaporated Au NPs. Owing to the hydrogen-bonded structure of DES, the size and morphology of the self-assembled Au films can be precisely controlled. The as-prepared 3D Au substrate exhibits outstanding SERS detection for various analyte molecules such as crystal violet (CV), rhodamine 6G (R6G), and 5,5′-dithiobis­(2-nitrobenzoic acid) (DTNB) with a limit of detection (LOD) as low as 3.2 × 10^–15^ M and exceptional SERS signal reproducibility. Moreover, the 3D Au-SERS substrate showed outstanding sensitivity in detecting a variety of target molecules, such as doxorubicin (DOX) with a LOD of 5.8 × 10^–12^ M and heavy metal ions such as As^3+^ and Hg^2+^ with LODs as low as 5.5 × 10^–5^ and 4.1 × 10^–5^ g/mL, respectively. Additionally, we validate their applicability in SERS detection of nanoplastics such as polyethylene terephthalate (PET), poly­(methyl methacrylate) (PMMA), and polystyrene (PS), offering a great potential in practical SERS detection of various environmental pollutants.

## Graphical abstract

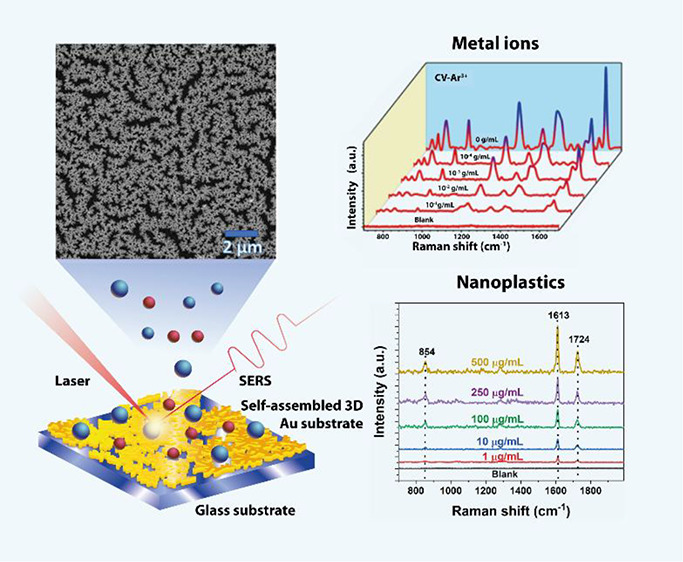

## Introduction

The surface-enhanced Raman scattering (SERS) spectroscopy technique has emerged as a highly powerful, label-free detection technique for ultrasensitive detection of chemical and biological probe molecules over plasmonic metal nanostructures through electromagnetic enhancement mechanisms.^1,2^ Current research efforts are mainly focused on developing novel methods to create ordered plasmonic metal nanoparticles (NPs), such as Ag, Au, and Cu, separated by narrow subnanometric gaps (less than 10 nm).^3–5^ These gaps can localize a strong electromagnetic (EM) field at the so-called “hotspots” created by localized surface plasmon resonances (LSPRs) of noble metal nanoparticles.^6–8^ Such nanometric gaps between plasmonic NPs generate a stronger electromagnetic field via the near-field coupling of LSPR, allowing significant Raman scattering signals when analyte molecules are placed near the nanometric gaps.^9–11^ However, precise control of these “hotspots” is a challenge, which affects the reproducible detection of analyte molecules at the single-molecule level.^8,12^

Three-dimensional (3D) porous plasmonic nanostructures of Au, Ag, and Cu with nanogaps/pores has gained significant attention for SERS detection of a variety of analyte molecules at different sizes.^13–16^ These gaps or microporous/nanoporous structures often exhibit a broad plasmonic absorption ranging from ultraviolet (UV) to near-infrared (NIR) regions. In addition, they exhibit an intense local electric field confinement within the nanopore sites, allowing a remarkable enhancement of Raman signal of analyte molecules that are close to the gaps and pores.^16,17^ Numerous bottom-up and top-down approaches including the dealloying process,^15^ electron-beam lithography or photolithography techniques,^18^ nanoimprint lithography,^19^ or the conventional nanoparticle colloidal self-assembly process^20^ have been adapted to create 3D porous or ordered structures. Although these techniques allow the creation of well-ordered plasmonic nanostructures with excellent control over shape and nanometric gaps, these techniques are limited for scalable preparation and require complicated reagents and expensive equipment. Recently, the vacuum thermal evaporation technique has been considered a low-cost and highly efficient method to grow high-purity metallic thin films on a solid substrate.^21^ Although this technique enables the creation of high-quality metallic smooth thin films, forming nanoparticles and controlling their self-assembly and surface structure to generate uniform gaps between adjacent nanoparticles are challenging. Therefore, various organic solvents or liquids have been used to deposit metallic/bimetallic NPs onto surfaces, effectively controlling their morphologies and self-assembly structures.

Recently, deep eutectic solvents (DESs) have shown promise as “green solvents” for nanomaterial synthesis and self-assembly of metallic NPs.^22–24^ DESs are generally formed by mixing hydrogen bond acceptors (HBAs), typically consisting of quaternary ammonium salt such as choline chloride (ChCl), and hydrogen bond donors (HBDs) consisting of a wide range of compounds such as carboxylic acids, polyols, and amides.^25,26^ In particular, DES formed by ChCl and urea is recognized as a commonly used combination because of its desirable properties such as good biodegradability, low toxicity, high viscosity, and ionic character.^27^ Importantly, the ChCl/urea-derived DES exhibits la ow melting point (285.85 K),^27^ high thermal stability (above 383.2 K),^28^ and extremely low vapor pressure in the range of 35.32 to 60.77 Pa below 343.15 K,^29^ making it ideal for vacuum deposition systems. These properties make ChCl/urea a particularly suitable solvent for the controlled growth and self-assembly of various metallic NPs. However, the integration of these DESs within vacuum conditions to grow and self-assemble metal NPs with controlled “hotspots” is highly challenging.

In this work, we demonstrate a green and low-cost synthesis method for the preparation of 3D Au films by using vacuum thermal evaporation onto the DES surface. The use of DES on the growth substrate facilitates controlled self-assembly during the vacuum thermal evaporation process. By variation of the deposition pressure, the self-assembly structure and interparticle gaps can be easily controlled. In particular, the dendritic-like 3D Au film possesses a high density of SERS “hotspots”. As a result, the fabricated SERS substrate exhibited outstanding sensitivity in the detection of different analyte molecules with LOD down to 10^–15^ M. Importantly, the fabricated SERS substrate has demonstrated exceptional sensitivity for the trace detection of the anticancer drug (doxorubicin (DOX)) and heavy metal ions (As^3+^ and Pb^2+^) as well as the identification of different nanoplastic particles such as PET, PMMA, and PS with low detection limit, offering the possibility for practical detection.

## Experimental Section

### Materials

Choline chloride (HOC_2_H_4_N­[CH_3_]_3_Cl, ≥98%), ethylene glycol (HOCH_2_CH_2_OH, anhydrous; 99.8%), malonic acid (CH_2_(COOH)_2_, 99%), and urea (NH_2_CONH_2_, 99.0%) were acquired from Merck Mexico. The gold wire (100%, 0.2 mm) was obtained from Spi Supplies. Crystal violet (CV, ≥98%), rhodamine 6G (R6G, ∼95%), 5,5′-dithiobis­(2-nitrobenzoic acid) (DTNB, ≥98%), methyl methacrylate (MMA, 99.0%), 2,2′-azobis­(2-methylpropionamide) (99.0%), trifluoroacetic acid solution (TFA, 90% v/v), sodium dodecyl sulfate (SDS, 99.0%), DOX, As^3+^, Hg^2+^, and bovine serum albumin (BSA) were purchased from Sigma Aldrich, Mexico. Acetone, trichloroethylene, and ethyl alcohol were obtained from J. T. Baker. Commercial polystyrene particles (PS, standard particle size of 100 nm) were purchased from Sigma Aldrich, Mexico. Deionized water (DI) was used for all experiments including washing cycles of the samples. Tap water samples were collected from the FCFM-BUAP building, and lake water samples were collected from the lake garden at CUBUAP campus.

### Preparation of Self-Assembled Au NP Films

Frosted glass slides employed in this study underwent thorough cleaning in acetone, trichloroethylene, and ethyl alcohol in an ultrasonic bath, each for 10 min. Subsequently, a deep eutectic solvent (DES) composed of ChCl and urea (U) in a 1:2 molar ratio was employed to coat the frosted glass surface. To prepare approximately 20 mL of DES, 11 g of ChCl and 9.4636 g of urea were used. The components were mixed and heated at 80 °C for 1 h under continuous stirring until a homogeneous and transparent eutectic mixture was obtained as reported elsewhere.^30^ Before the thermal evaporation of Au, 1 mL of DES was uniformly applied onto ground glass slides over an area of 2.5 × 2.5 cm^2^, corresponding to an application density of 0.16 mL/cm^2^.

To obtain Au NPs films, a Au nanowire (approx. 31.3 mg) was thermally evaporated and deposited onto the DES-coated surface at various pressures: P1 = 1 × 10^–2^ mbar, P2 = 1 × 10^–3^ mbar, P3 = 1 × 10^–4^ mbar, and P4 = 2 × 10^–4^ mbar. All samples were subjected to a 4 A current supplied to the tungsten filament during deposition. Following deposition, the Au NP film was transferred to another glass substrate by forming a sandwich structure (Figure 1a). Finally, the adhered Au films were washed using DI water to eliminate DES residues, and then the samples were dried at room temperature for 6 h.

**Figure 1:**
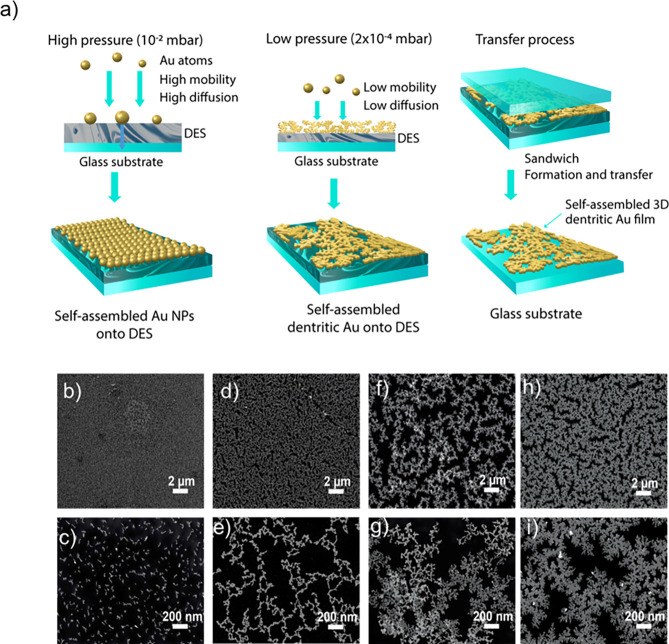
(a) Schematic illustration of the synthesis steps involved in the self-assembly and formation of branched Au films. (b–i) Typical low- and high-magnification FE-SEM images of self-assembled Au films obtained at different deposition pressures: (b, c) 1 × 10^–2^, (d, e) 1 × 10^–3^, (f,g) 1 × 10^–4^, and (h, i) 2 × 10^–4^ mbar.

### Preparation of Nanoplastic Particles

PET nanoplastics, with an average size ranging from 50 to 300 nm, were synthesized following a previously established protocol.^40^ Specifically, 1 g of PET particles underwent complete dissolution after agitation for 2 h at 50 °C in 10 mL of concentrated trifluoroacetic acid solution (TFA, 90% v/v). The solution was then stored overnight. Nanoplastic particles were precipitated from the solution by adding 10 mL of diluted TFA (20% v/v) while vigorously swirling the mixture for 2 h. To isolate the nanoplastic particles, the suspension underwent centrifugation at 2500 rpm for 1 h. Subsequently, the pellet was redissolved in 50 mL of 0.5% sodium dodecyl sulfate (SDS), vigorously agitated, and sonicated to create a homogeneous solution.

The preparation of poly­(methyl methacrylate) (PMMA) nanospheres was carried out by the chain-growth method as reported earlier.^31^ In a typical procedure, 1600 mL of DI water was mixed with 300 mL of methyl methacrylate (PMMA) solution in a three-neck flask, and then nitrogen was purged to create an inert atmosphere. The solution mixture was heated to 70 °C under magnetic stirring (350 rpm). When the temperature had stabilized, the nitrogen was turned off, and then 1.5 g of 2,2′-azobis­(2-methylpropionamide) was introduced into the flask. After 60 to 90 min, the solution mixture turned into a white colloidal suspension, confirming the formation of PMMA nanospheres. Plastic nanoparticles were subjected to multiple rinsing cycles using DI water during the purification and extraction processes.

### Characterization

Images were acquired using a JEOL-JSM7401F field emission scanning electron microscope (FEDSEM) operating at 15 kV. Topography images were obtained using the XE-7 Park Systems atomic force microscopy (AFM) instrument (Park Systems Corp., Suwon, Korea). Optical absorption measurements for all nanoparticle samples were recorded across the wavelength range of 200–1150 nm using a StellarNet BLUE-Wave miniature spectrometer. SERS spectra were measured with a Bruker (SENTERRA) Raman spectrometer utilizing a 633 nm He–Ne laser as the excitation source with a laser power of 1.6 mW. The diameter of the exciting laser spot was consistently set at 4.7 μm under a 50× lens, and the signal acquisition time was fixed at 1 s for three cycles. The SERS imaging was carried out over a 20 × 20 μm^2^ area, with a step size of 0.5 μm.

### Finite Difference Time Domain (FDTD) Simulation of Au NP Films

The structural parameters for the FDTD model were derived from Au nanoparticle atomic force microscopy (AFM) and scanning electron microscopy (SEM) images. The simulation region was established at 1000 × 1000 nm^2^, and the refractive index of the surrounding medium was set to 1.0 to replicate air conditions. The simulation mesh was set to 2 nm to ensure precision. For Au nanoparticles, the total simulation period extended to 250 ns, covering the wavelength range of 400–900 nm. The FDTD solutions software (Lumerical Inc., Canada version) was employed to simulate the electromagnetic field distribution in the samples. The dielectric constant value of Au, crucial for these simulations, was obtained from the CRC Handbook of Chemistry and Physics through the software’s material database. The simulations employed a total-field/scattered-field (TFSF) source with perfectly matched layer (PML) boundary conditions.

### SERS Detection

For the detection of analyte molecules, the 3D Au substrates were coated with a 25 μL (10^–6^ M) aqueous solution of crystal violet (CV), rhodamine (R6G), and 5,5′-dithiobis­(2-nitrobenzoic acid) (DTNB) molecules with different concentrations followed by a 3 h drying period at room temperature before acquiring the SERS spectrum. For SERS detection of DOX, the aqueous solution of bovine serum albumin (BSA) containing DOX (5 mg/mL) was prepared and diluted into different concentrations from 10^–8^ to 10^–12^ M. Then, 25 μL of solution was deposited onto the 3D Au substrate and allowed to dry overnight at ambient conditions. For SERS detection of heavy metal ions such as arsenic (As^3+^) and mercury (Hg^2+^), the metal ions with varied concentrations were mixed with a fixed concentration of CV (10^–6^ M) followed by 25 μL of the BSA solution being deposited over the SERS substrate, and the mixture was allowed to naturally dry before the SERS measurements. For nanoplastic detection, the as-prepared PET, PMMA, and commercial PS (100 nm size) nanoparticles were dispersed in DI water at various concentrations. Then, the fabricated 3D Au substrates were coated with different solutions containing 25 μL of nanoplastic particles, and the substrates were left to dry at room temperature for 3 h. SERS detection was then performed on the substrates. For identification of nanoparticles in natural water conditions, a similar method was applied by spiking nanoplastic particles in tap and lake water and then depositing onto the SERS substrate.

## Results and Discussion

The experimental sequence detailing the DES-mediated self-assembly of Au NPs to produce extensive self-assembled porous Au films is outlined in Figure 1a. Initially, the DES is deposited onto glass substrates and positioned inside of the thermal evaporation chamber. Subsequently, Au wires are thermally evaporated using a tungsten filament by applying a current of 8 A. The resulting evaporated Au metal atoms are then deposited over the DES-coated surface. Importantly, the DES solvent remains stable and does not evaporate during thermal deposition, demonstrating excellent stability over the glass substrate. Following the deposition, the Au NPs over the DES-coated surface are transferred into another glass substrate by creating a sandwich structure. Finally, subsequent washing removes the DES from the substrate (Figure 1a), then it is left to dry under ambient conditions, yielding self-assembled Au NP films over a large area. Notably, without DES coating, the self-assembled Au NPs were not observed; instead, a smooth, uniform Au film was obtained (Figure S 1, Supporting Information).

The field-emission scanning electron microscopy (FE-SEM) images in Figure 1b–i show the self-assembled Au NP films produced under different vacuum pressures during the thermal evaporation process. In Figure 1b,c**,** the Au films deposited at a higher pressure (P1, 1 × 10^–2^ mbar) exhibit dense, nearly spherical Au NPs uniformly arranged to form a compact Au NP monolayer film. Upon lowering the pressure (P2, 1 × 10^–3^ mbar), the initially dense Au NP films transform into a self-assembly of Au NPs, forming a distinctive one-dimensional chain-like structure, as depicted in Figure 1d,e. With further decrease in deposition pressures (P3 and P4; 1 × 10^–4^ and 2 × 10^–4^ mbar), the one-dimensional self-assembled networks actively evolve into dendritic-shaped Au nanostructure films as shown in Figure 1 f-i. As shown in Figure 1h,i, the Au films obtained at lower pressure (P4, 2 × 10^–4^ mbar), with dendritic-like Au nanostructures, self-assemble into a 3D interconnected network structure with nanometric gaps. These outcomes signify that lowering the pressure is a requisite for achieving a 3D dendritic Au nanostructure film, resulting in the generation of uniform and high-density “hotspots” crucial for SERS signal enhancement.^32^ Importantly, the self-assembled Au films for large areas can be easily obtained (Figure S2). The atomic force microscopy (AFM) results also confirmed that Au films formed under different deposition pressures have uniform film thickness and different morphologies, which align qualitatively with images obtained from SEM results. The relative height between the substrate and the self-assembled Au NP film is determined to be approximately between 5- 10 nm, as indicated by the height distribution profiles presented in the insets of each AFM image (Figure S3).

The optical absorption characteristics of the obtained Au films under different pressures were monitored by using UV–vis absorption spectroscopy (Figure 2a). The Au NP films acquired at higher pressures (Au-P1 and Au-P2) exhibited two distinct localized surface plasmon resonance (LSPR) peaks at 550 and 840 nm arising from the SPR of self-assembled Au NP aggregates.^33,34^ Conversely, in samples obtained at low pressures (Au-P3 and Au-P4), the two SPR peaks experienced a red shift toward higher wavelength regions. Notably, the intensity of the SPR peak at 850 nm increased for Au-P3 and Au-P4 films, which could be attributed to the formation of a branched dendritic Au through the self-assembly.^33^

**Figure 2:**
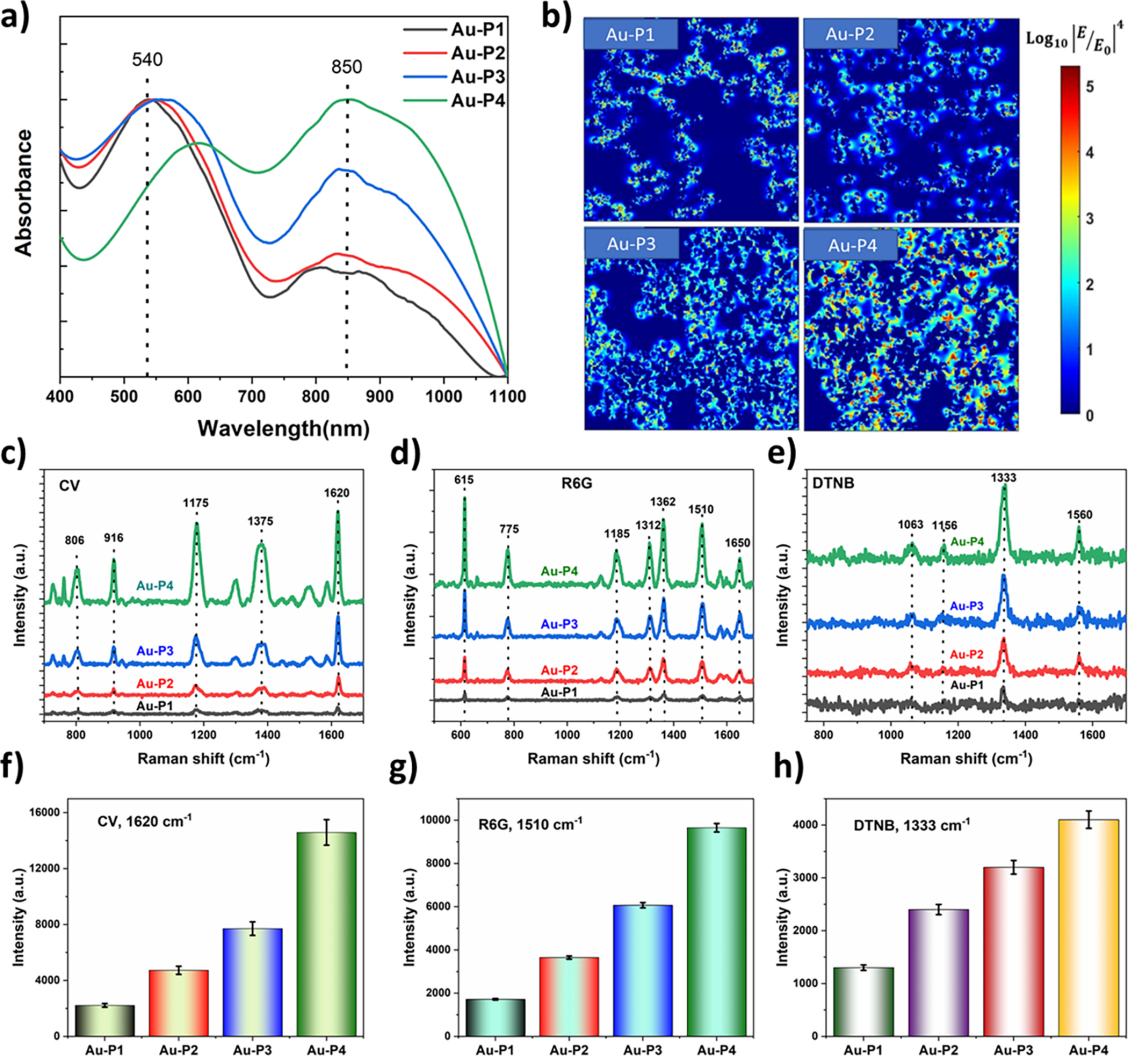
Optical and SERS properties of the self-assembled Au NP films obtained at different thermal evaporation pressures. (a) UV–vis spectra, (b) FDTD simulation, and (c–h) SERS performance of Au NP films obtained at different pressures (P1 = 1 × 10^–2^, P2 = 1 × 10^–3^, P3 = 1 × 10^–4^, and P4 = 2 × 10^–4^ mbar).

To unveil the electric field distribution within the self-assembled Au films, numerical simulations were conducted by using the finite-difference time-domain (FDTD) method. The electric field distribution arising from the plasmonic interaction of light with nanostructures was obtained at 633 nm by using an X-polarized plane wave. As shown Figure 2b, the dendritic Au film (Au-P4) exhibited a more robust electric field distribution at the tips and gaps compared with the other films obtained at higher pressures (Au-P1, Au-P2, and Au-P3). Additionally, it was evident that the laser excitation wavelengths of 633 and 785 nm exhibited a stronger EM field concentrated at the gap sites compared with 532 nm (Figure S4), which could be attributed to the resonance between the LSPR peaks with the laser excitation wavelengths. Notably, such strong EM field distribution of Au-P4 films is induced by the dendritic morphology assembling into a dense compact structure with small gaps, generating the maximum dense SERS “hotspots”. The overall theoretical enhancement factor (EF) was approximately proportional to the fourth power of the enhancement of the local electric field by the following relation: *E*_SERS_ = |*E*_max_/*E*_0_|^4^, where *E*_max_ and *E*_0_ are the maximum and the incident local EM field, respectively.^9^ The theoretical SERS enhancement factor (EF) is estimated for the 3D dendritic Au NP films to be ∼9.8 × 10^5^, 2 orders higher than spherical Au NPs obtained at higher deposition pressures.

### SERS Detection Performance of 3D Au-SERS Substrate

To quantitatively assess the SERS performance of the self-assembled Au films, SERS measurements were conducted for molecular detection; crystal violet (CV, 10^–6^ M), rhodamine 6G (R6G), and 5,5′-dithiobis­(2-nitrobenzoic acid) (DTNB) molecules were utilized as a probe analyte molecule on the substrate. First, the SERS activity of the Au films was investigated to determine the optimal deposition pressure. Figure 2c–e presents the Raman spectra of CV, R6G, and DTNB (10^–6^ M) molecules onto Au substrates obtained at different thermal evaporation pressures. As depicted in Figure 2c, the SERS spectra of CV display characteristic peaks at 806 and 1172 cm^–1^ attributed to the in-plane bending vibration of C–H, while the peaks at higher wavelengths, 1585 and 1616 cm^–1^, are ascribed to the stretching of ring C–C.^35^ The SERS spectra of R6G in Figure 2d exhibit peaks at 613, 773, and 1126 cm^–1^ arising from the in-plane and out-plane bending of the C–C–C ring and C–H in-plane bending vibrations, respectively. Other peak positions at 1184, 1362, 1507, and 1649 cm^–1^ are associated with the symmetric modes of in-plane C–C stretching vibrations.^36^ Also, the SERS spectra of DTNB displayed characteristic peaks at 1333, 1067, 1152, and 1558 cm^–1^ (Figure 2e), which are attributed to the symmetric stretching vibrations of the nitro groups of DTNB, the succinimidyl N–C–O stretching overlapping with aromatic ring modes, the C–H deformation modes, and the aromatic ring C–C stretching modes, respectively.^37^ As can be seen from Figure 2f–h, the 3D dendritic Au films (Au-P4 sample) exhibited a higher SERS signal intensity at the most prominent peaks of CV (1620 cm^–1^), R6G (1510 cm^–1^), and DTNB (1340 cm^–1^), respectively. Notably, the 3D Au substrate can enhance the SERS signal of CV molecules (10^–6^ M) by about 27-fold higher compared with the non-SERS conditions (Figure S5). The SERS enhancement factor (EF) was quantitatively estimated using the previously reported procedure (see the Supporting Information).^14^ The calculated SERS EFs are about 2.99, 1.42, and 0.82 × 10^4^ for CV, R6G, and DTNB using the 3D dendritic Au substrate (Au-P4), which are much greater compared with the other Au substrates obtained at higher pressures (Table S1).

The detection sensitivity of the substrate was meticulously probed by varying the concentration of the analyte molecules such as CV, R6G, and DTNB molecules (Figure 3a–c). As can be noticed from Figure 3a–c, the Raman intensity of all three analyte molecules exhibits a gradual decrease with the lowering concentration, and even at lower concentrations, the peaks were detected, emphasizing the superior sensitivity of the SERS substrate. In addition, the prominent Raman intensity of CV at 1620 cm^–1^, R6G at 1510 cm^–1^, and DTNB at 1333 cm^–1^ showed a linear correlation in the measured concentration range (Figure 3d–f), which is indicative of the substrate’s remarkable sensitivity and the feasibility of quantitative detection of analyte molecules over a wide concentration range. The limit of detection (LOD) is calculated using the following equation:^38^ $\mathrm{LOD}=\frac{3\sigma }{m}$ , where σ is the standard deviation of the SERS intensity of the blank measurements, while *m* represents the slope of plotted calibration curves. The estimated LOD of the 3D dendritic Au-SERS substrate is about 3.2 × 10^–15^, 1.2 × 10^–1^ , and 1.6 × 10^–15^ M for CV, R6G, and DTNB, respectively.

**Figure 3:**
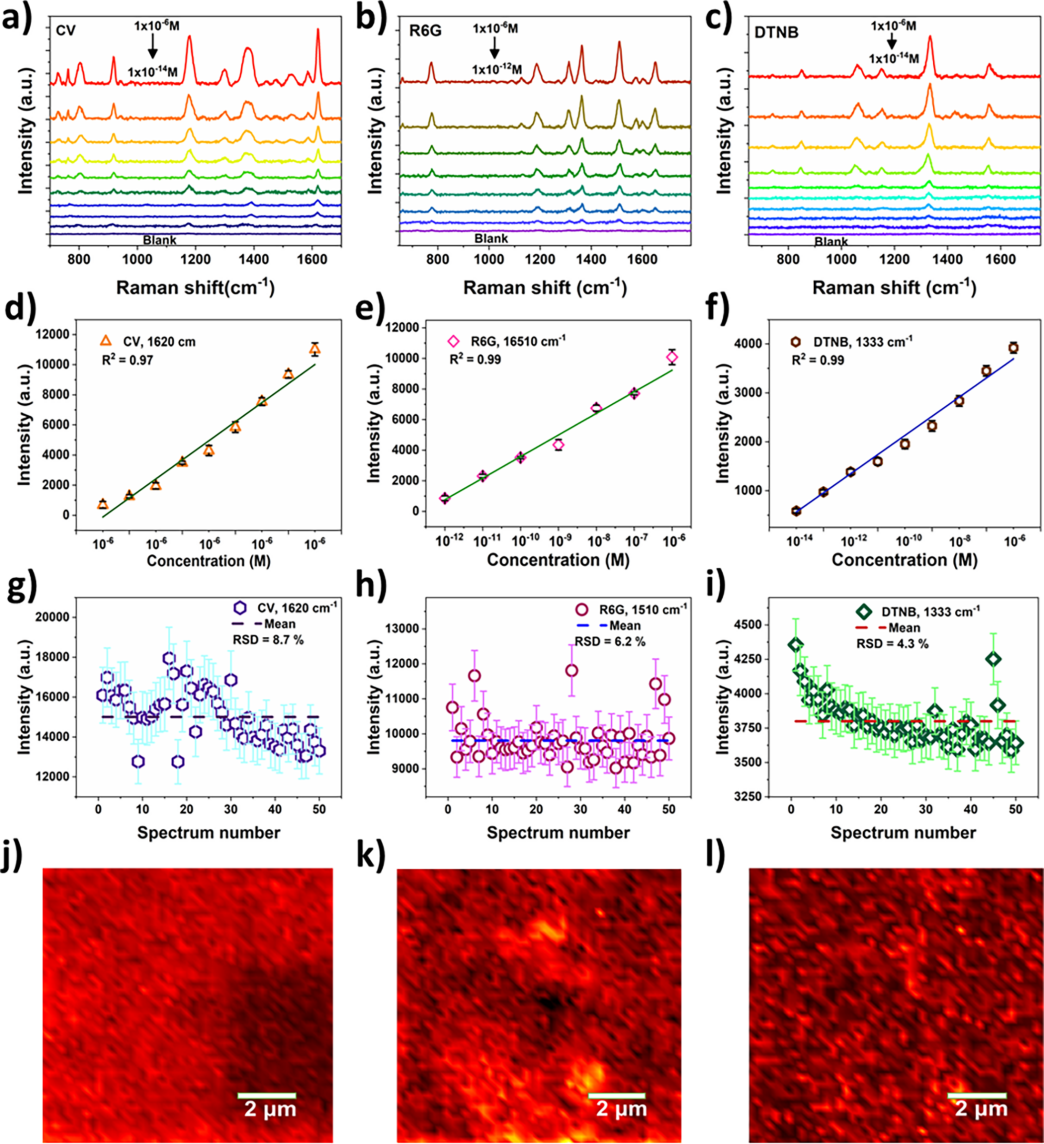
SERS sensitivity and reproducibility characterization of 3D Au film substrates. (a–c) Concentration-dependent SERS spectra of (a) CV, (b) R6G, and (c) DTNB molecules. (d–f) Calibration plot showing the peak intensity at 1620 cm^–1^ (CV), 1510 cm^–1^ (R6G), and DTNB (1333 cm^–1^) as a function of concentration. (g–i) Relative standard deviation from 50 Raman spectra measured on porous the Au film substrate. (j–l) SERS mapping images of CV, R6G, and DTNB (10^–6^ M) onto the 3D Au-SERS substrate.

In addition to high sensitivity, the SERS signal reproducibility was evaluated by acquiring SERS spectra from 50 distinct spots within the same substrate (size: 2 × 2 cm^2^) and calculating the relative standard deviation (RSD) of the SERS prominent peak intensity of respective analyte molecules. The SERS intensity of 10^–6^ M of CV, R6G, and DTNB across these 50 regions remains nearly identical, affirming that the substrate has high reproducibility (Figure S6). The calculated results showed that their RSD values are 8.71 CV, 6.2 (R6G), and 4.3% (DTNB) for the prominent peak of CV, R6G, and DTNB, respectively, which are significantly lower than those of reported commercial Au NP-based SERS substrates (RSD ≈ 15–20%).^39–41^ Specifically, the DTNB analyte over the 3D substrate showed a much lower RSD compared with the other analyte molecules, which can be attributed to the higher adsorption of DTNB molecules over the metallic substrates.^42^ In addition, the SERS substrate showed excellent stability as it retains about 70.57% after a 20 day period (Figure S7). On the other hand, the SERS mapping images of the three analyte molecules over the 3D dendritic Au substrate with a size of 10 × 10 μm^2^ area (Figure. [Fig fig3]j–l) revealed the relatively uniform distribution of analyte molecules over the entire mapping region, further confirming the high uniformity over a large area. Notably, the SERS mapping image of multiplex analyte molecules can be easily obtained (Figure S8).

To demonstrate application of the fabricated 3D Au substrate, first we evaluated the SERS detection of anticancer drugs such as DOX. DOX is the most commonly applied chemotherapy drug belonging to the anthracycline’s family, which is widely employed to the treatment of a broad range of human malignancies.^43^ For SERS detection of DOX, the BSA solution was used (more details in the Experimental Section). Figure 4a shows the SERS spectra of DOX with varied concentrations ranging between 10^–8^ to 10^–11^ M in BSA. It is evident from Figure 4a that SERS spectra show the characteristic vibrational peak at 840 cm^–1^ and an intense peak at 1233 cm^–1^, which are assigned to the C–C and C–O stretching vibrations of DOX drug molecules.^44^ The SERS signal of DOX was clearly observed even at a concentration of 1 × 10^–11^ M. The LOD was estimated to be about 5.8 × 10^–12^ M, which is much lower than the therapeutic window of DOX (10 to 80 nm).^44^ Notably, a linear trend was observed for the SERS peak intensity of DOX (peak at 1233 cm^–1^) as a function of concentration (Figure 4b), indicating the feasibility of quantitative detection of DOX drug molecules over a wide concentration range.

**Figure 4:**
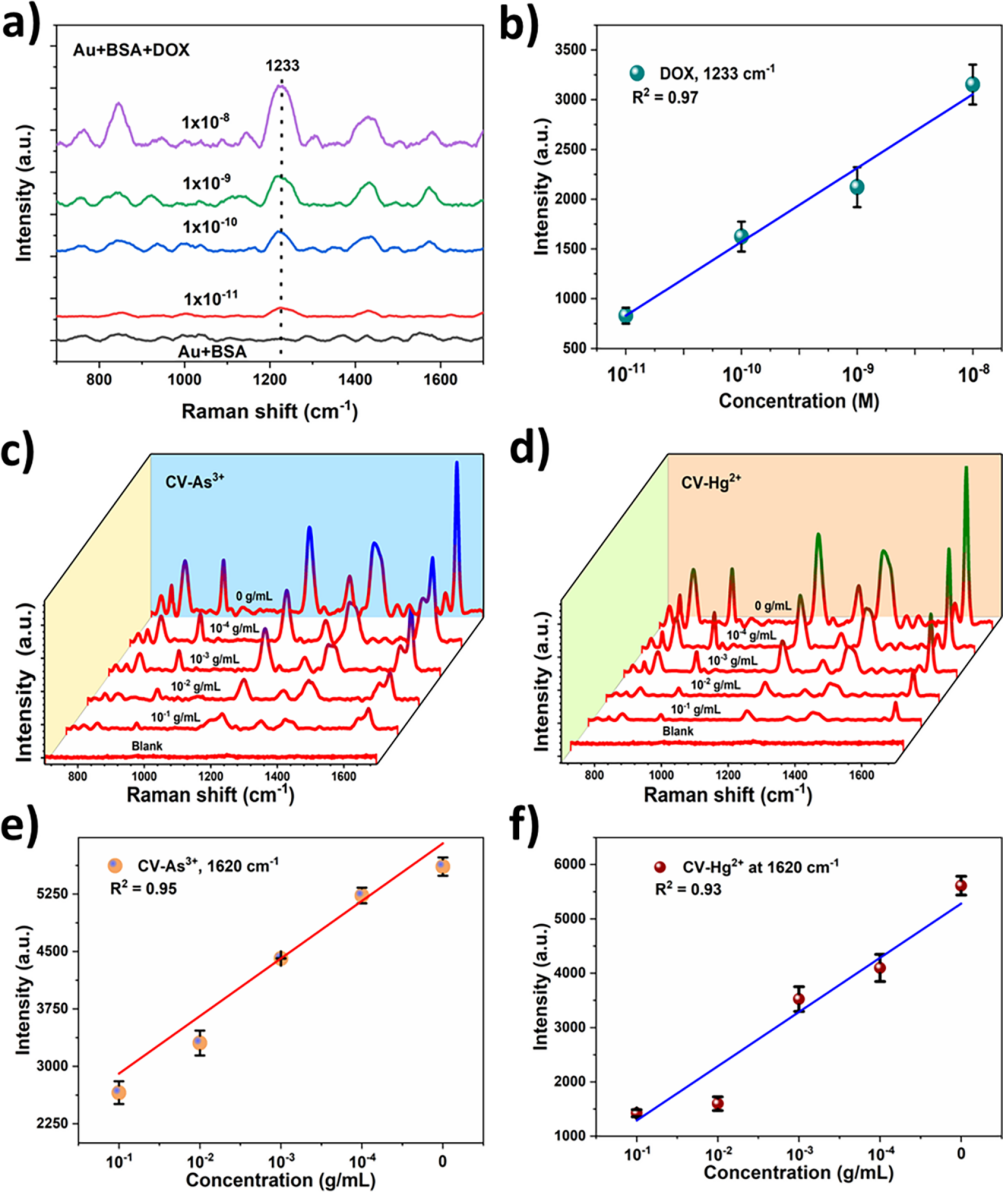
SERS detection performance of the 3D Au substrate for anticancer drug heavy metal ion sensing: (a, b) SERS spectra of DOX with different concentrations and corresponding linear calibration plot, (c, d) SERS spectra of CV mixed with different concentrations of As^3+^ and Hg^2+^, and (e, f) corresponding linear calibration plots.

In addition, the 3D Au substrate was utilized for SERS detection of toxic heavy metal ions such as As^3+^ and Hg^2+^. It was well-known that the Raman inactive analytes such as metal ions cannot be detected directly by SERS. However, previous studies have demonstrated that upon the addition of metal ions with the SERS active probe molecules such as CV or R6G molecules, the SERS peak intensity was found to weaken due to their interaction and replacement of analyte molecules with the metal ions.^45,46^ Thus, we used CV (10^–6^ M) as the probe molecule for the detection of As^3+^ and Hg^2+^ions. Figure 4c,d shows the SERS spectra of only CV (10^–6^ M) coated Au substrate and As^2+^ and Hg^2+^ with different concentrations ranging between 10^–1^ and 10^–4^ g/mL. The SERS peak intensity of the CV molecules gradually weakens upon increasing the concentration of As^3+^ and Hg^2+^. Moreover, the characteristic peak intensity of CV at 1620 cm^–1^ as a function of As^3+^ and Hg^2+^concentration exhibited a linear correlation (Figure 4e,f), suggesting that the 3D Au-SERS substrate is capable of performing quantitative detection of metal ions. The estimated LOD is about 5.5 × 10^–5^ and 4.1 × 10^–5^ g/mL for As^3+^ and Hg^2+^P, respectively.

Finally, the application of the as-fabricated 3d Au film substrates for SERS detection of environmental pollutants such as nanoplastic particles, PET, PMMA, and commercial PS was explored (see experimental details). Figure 5a–c andFigure S9 show the SEM images of nanoplastic particles supported over the 3D Au substrate. The results revealed that PET, PMMA, and PS were distributed onto the 3D Au substrate with an average size of 764 ± 154, 417 ± 15, and 110 ± 13.2 nm, respectively. Notably, the PMMA and PS nanospheres are found in the gap’s sites, and PET nanoplastics are over the 3D Au substrate, due to the low particle size. As depicted in Figure 5d–f, the SERS spectra showed a characteristic peak for each type of nanoplastic with varied concentrations. Specifically, the PET particles exhibited distinct peaks at 864 cm^–1^ (ester C­(O)O bending mode), 1613 cm^–1^ (aromatic C–O stretching vibration), and 1724 cm^–1^ (C=O stretching vibration);^47,48^ PMMA showed peaks at 811 cm^–1^ (C–O stretching) and 1452 cm^–1^ (C–H bending);^49^ and PS displayed a peak at 1002 cm^–1^ (ring vibrating modes monosubstituted aromatic compounds or C–C ring breathing) and 1031 cm^–1^ (C–H vibration).^41,50^ Notably, the prominent peak in SERS spectra of each nanoplastic at various concentrations follows the linear trend with decreasing the concentration (Figure 5g–i), indicating the capability of the quantitative detection of nanoplastics over a wide concentration range. The LOD of the SERS substrate was calculated to be about 1.3 × 10^–7^ g/mL for PET, 4.5 × 10^–6^ g/mL for PMMA, and 6.8 × 10^–7^ g/mL for PS nanoplastic particles, respectively. The above results showed that in comparison with the smaller PS nanoplastics, PET and PMMA exhibited high sensitivity, which could be due to the low scattering cross section of PS nanospheres within the pores of the 3D dendritic Au substrate, as observed by many previous works.^49,50^ The observed LOD for nanoplastic detection is much lower compared with the state-of-the-art SERS substrates based on plasmonic nanostructures demonstrated previously (Table S2). The Raman enhancement of the nanoplastics could be attributed to the following factors: (1) The nanoplastic particles of smaller size are trapped inside the “hotspots” sites, thereby enhancing the near-field SERS signals.^51,52^ (2) The larger nanoplastic particles over the 3D Au substrate can also enhance the far-field SERS intensities since the 3D Au substrate consists of abundant and easily accessible “hotspots”.^53^ Thus, the SERS signal enhancement in nanoplastic particles of different sizes onto the 3D SERS substrate depends on the position and size of nanoplastic particles measured onto the substrate.

**Figure 5:**
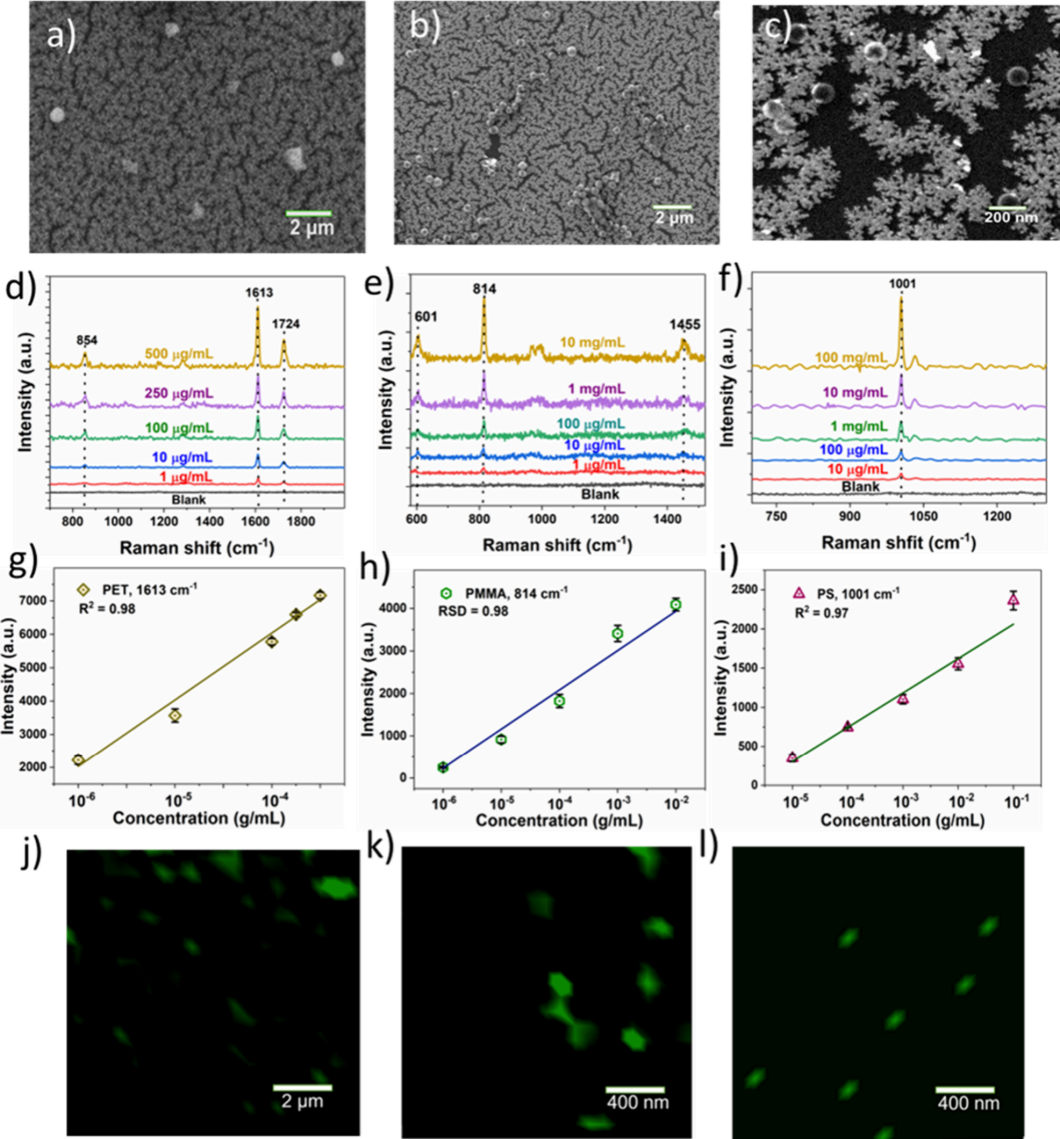
SERS detection and imaging of nanoplastics using a 3D Au substrate. (a–c) SEM images of of (a) PET, (b) PMMA, and (c) PS nanoplastics onto 3D Au substrate; (d–f) SERS spectra for PET, PMMA, and PS; (g–i) corresponding calibration curves; (j–l) SERS mapping images of PET, PMMA, and PS onto 3D Au substrate over an area of 10 × 10 μm with a scanning step size of 0.1 μm, respectively.

SERS mapping also enables chemical identification on a selected area to identify the nanoplastic size, type, and composition on the surface of the SERS substrate with high resolution.^54,55^ Figure 5j–l reveal that the SERS color mapping images of three respective nanoplastics have a relative uniform dispersion in the 3D Au substrate for an area of 10 × 10 μm^2^. Specifically, the nanoplastics’ position and size can be effectively identified. Moreover, the SERS mapping of mixed nanoplastics over the substrate can easily be obtained (Figure S10). These results suggest that the 3D Au substrate enables the precise identification and mapping of various types of nanoplastics without a series of complicated separation process.

### Detection of Nanoplastic Particles in Real Water Samples

The accurate detection of nanoplastic particles in real-world systems remains a daunting challenge due to the presence of environmental impurities. To demonstrate the applicability of the 3D Au-SERS substrate, we carried out nanoplastic detection in natural water samples. For this, the PET, PMMA, and PS nanoplastic particles are dispersed in tap water and lake water and deposited onto the 3D Au substrate for detection (more details in the Experimental Section). As can be seen from Figure S11, the spectra of the PET, PMMA, and PS nanoplastic particles in tap water and lake water have an almost similar pattern compared to DI water. In the case of PMMA and PS nanoplastic particles, the SERS spectra consist of some noise, which could be attributed to the interference due to the interaction between nanoplastics and biological entities in tap and lake water.^49^ Importantly, the LOD for nanoplastic particles is observed in real water samples compared with that of DI water (Table S3). Thus, the 3d Au substrate showed a great potential for identifying multiple analyte molecules such as DOX, heavy metal ions, and nanoplastic particles in complex environmental conditions.

## Conclusions

In summary, we demonstrated a low-cost method for fabricating 3D Au films with unprecedented control over “hotspots” that offer highly sensitive SERS detection of variety of target molecules. Utilizing the distinct properties of DES, precise control of self-assembly structures was achieved by controlling the deposition parameters. The as-fabricated 3D Au substrate exhibited high SERS sensitivity for detecting a variety of analyte molecules such as CV, R6G, and DTNB molecules with LOD as low as 10^–15^ M concentration and exceptionally high signal reproducibility. Importantly, the 3D dendritic Au-SERS substrate has been utilized for the detection of multiple analyte molecules such as anticancer drugs (DOX) and heavy metal ions (As^3+^ and Hg^2+^) as well as nanoplastics (PET, PMMA, and PS) with superior sensitivity. This strategy offers a low-cost and scalable fabrication of 3D plasmonic films that could be easily transferred onto different flexible substrates, paving the way for designing efficient SERS substrates for the sensitive detection of a diverse range of environmental pollutants.

## Supplementary Material



## Notes

SEM images of Au NP films, Au film without using DES, and Au films deposited over different DESs; details of SERS-EF calculations; reproducibility SERS spectra of CV, R6G, and DTNB for 50 different sites; SERS mapping images of multiplex imaging; SEM and nanoplastics over the 3D Au substrate; comparison of SERS spectra obtained with the 3D Au substrate with the Au NP monolayer; and table summarizing the comparison of SERS detection performance of nanoplastics with previous studies (PDF)
