# Supplementary material for: Self-Assembled Three-Dimensional Au Films as Highly Reproducible and “Hotspots”-Rich Substrates for Multiplex SERS Detection
Source: Anal Chem. 2025 Aug 14;97(33):17982–91. doi: 10.1021/acs.analchem.5c01716 (PMC12392253; doi:10.1021/acs.analchem.5c01716)
Supplement: Supplementary file 1 [file ac5c01716_si_001.pdf]

Supporting information for:

Self-Assembled Three-Dimensional Au Films as Highly Reproducible and ″Hotspots″-Rich Substrates for Multiplex SERS-Detection

Rafael Villamil Carreón,[ll] *José Juan. Gervacio-Arciniega,*[§] *Ma. Estela Calixto,*[†] *Siva Kumar Krishnan*[#,] *

[ll]Facultad de Ciencias Físico Matemáticas, Benemérita Universidad Autónoma de Puebla, Av. San Claudio y Blvd. 18 Sur, Col. San Manuel, Ciudad Universitaria, Puebla, Pue. 72570, México

[§]SECIHTI-Facultad de Ciencias Físico Matemáticas, Benemérita Universidad Autónoma de Puebla, Av. San Claudio y Blvd. 18 Sur, Col. San Manuel, Ciudad Universitaria, Puebla, Pue. 72570, México

[†]Instituto de Física "Ing. Luis Rivera Terrazas", Benemérita Universidad Autónoma de Puebla, Av. San Claudio y Blvd. 18 Sur, Col. San Manuel, Ciudad Universitaria, Puebla, Pue. 72570, México

[#]SECIHTI - Instituto de Física "Ing. Luis Rivera Terrazas", Benemérita Universidad Autónoma de Puebla, Av. San Claudio y Blvd. 18 Sur, Col. San Manuel, Ciudad Universitaria, Puebla, Pue. 72570, México

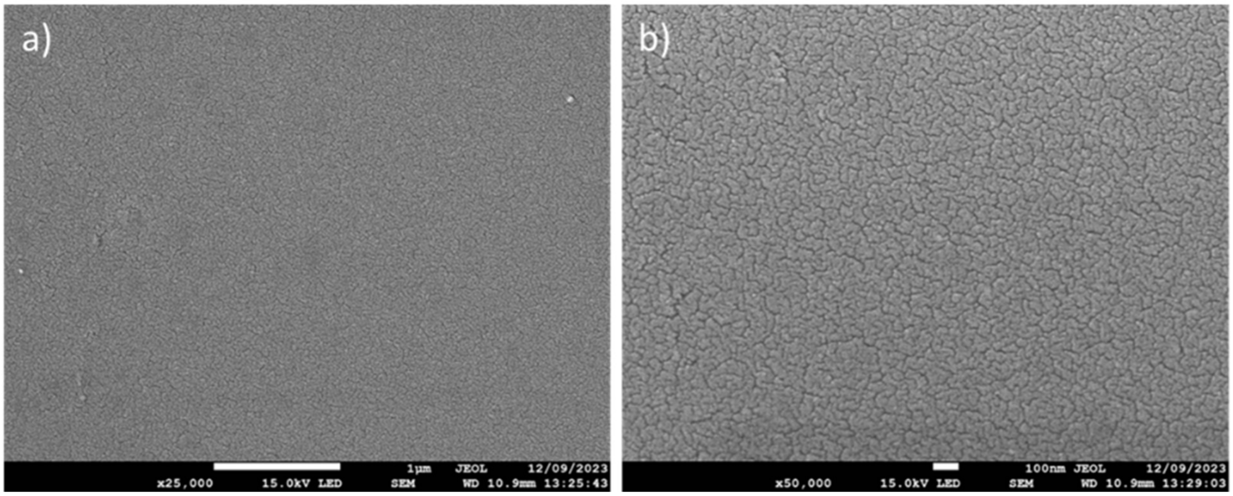

Figure S1. a, b) Typical SEM images of Au films obtained without utilizing DES onto growth substrate (glass). Deposition pressure of $2x10^{-4}$ mbar, applied current of 8 A.

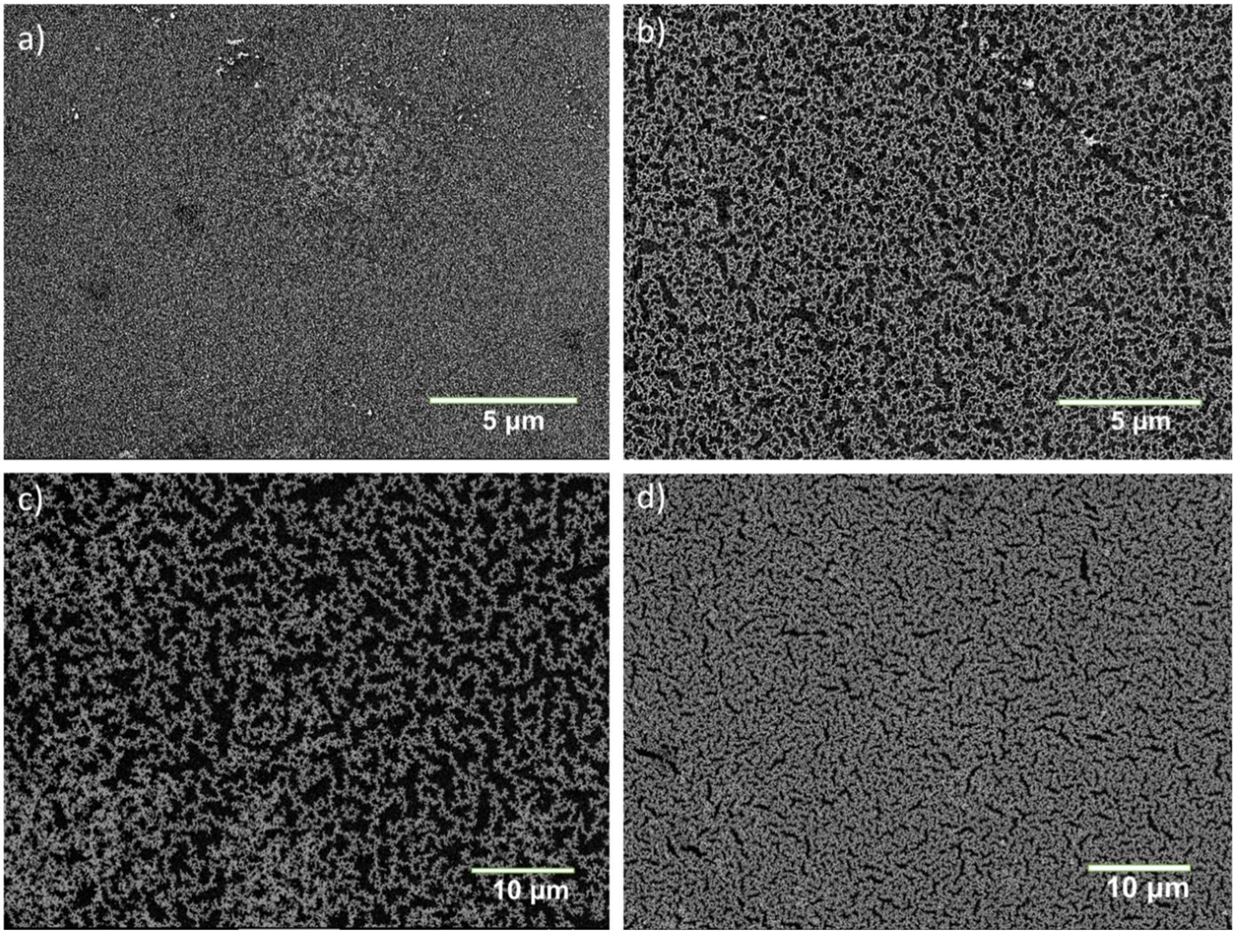

Figure S2. a-d) Low magnification SEM images of self-assembled Au NPs films through thermal evaporation onto DES-coated glass surface under different pressure. a) 10^2, b) 10^3, c) $1x10^4$ and d) $2x10^{-4}$ mbar, respectively.

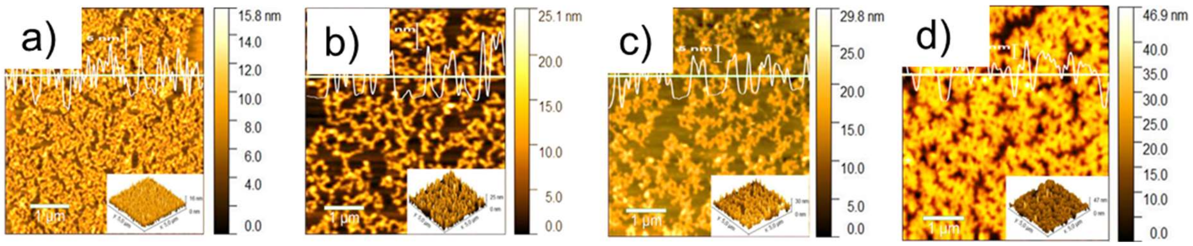

Figure S3. a-d) Typical AFM topographical images of Au films obtained at different thermal evaporation pressures. a) 10^2, b) 10^3, c) $1x10^4$ and d) $2x10^{-4}$ mbar, respectively.

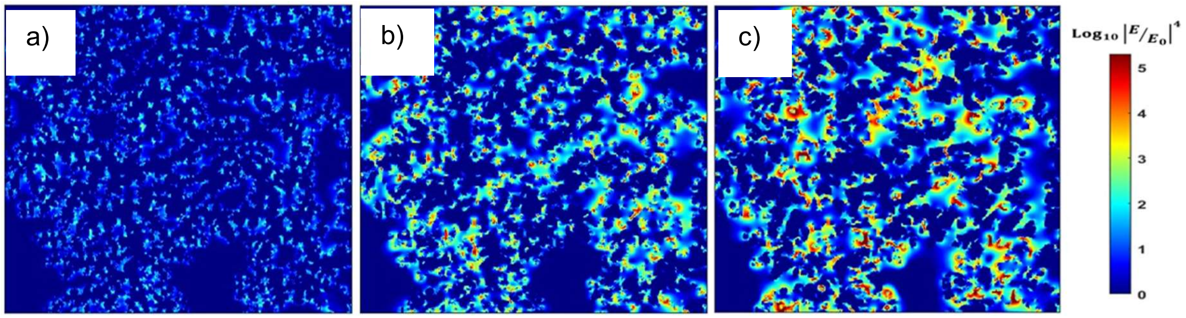

Figure S4. a-c) FDTD simulation of models of porous Au NPs films irradiated by an incident plane wave with different wavelengths a) 532, b) 633 and c) 785 nm, respectively.

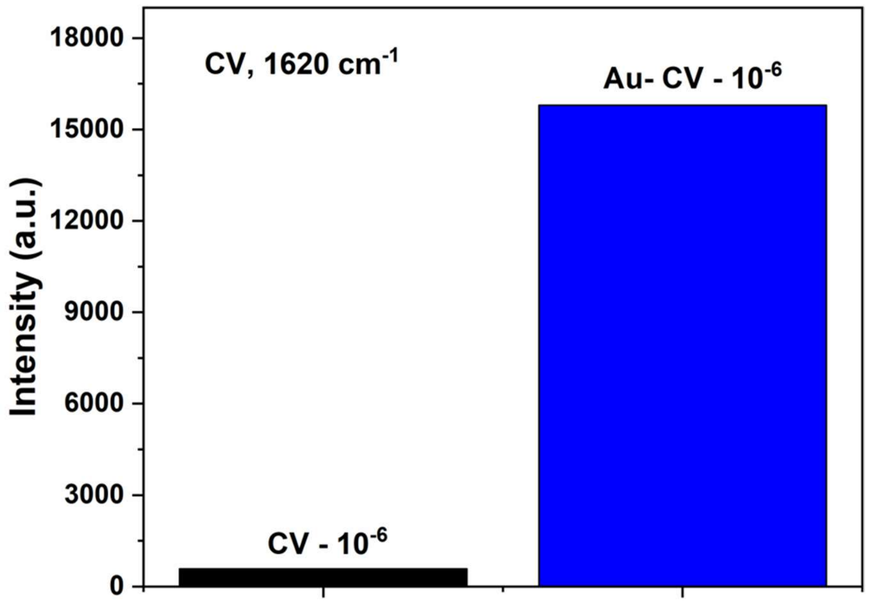

Figure S5. Comparison of the SERS performance of the CV molecules (10^{-6} M) over 3D Au substrate with the non-SERS (glass substrate).

SERS enhancement factor estimations:

To calculate the theoretical enhancement factor (EF) using the FDTD software, it is possible to implement an interface compatible with MATLAB to apply the theoretical $|E|^4$ approximation to the simulated electromagnetic field distribution. This approach allows for the identification of the maximum EF around the gold nanostructures. The theoretical equation was applied directly, without modifications or additional assumptions, using only the electric field distribution data obtained from the FDTD simulations to compute the SERS EFs.

The experimental SERS EFs was estimated using the following equation without modification.[1]

$$EF = \frac{(I_{SERS}/N_{SERS})}{(I_{ref}/N_{ref})}$$

Where:

- I_{SERS}: Raman intensity of the analyte on the SERS substrate.
- I_{ref}: Raman intensity of the analyte under non-SERS (reference) conditions.
- N_{SERS}: Number of molecules contributing to the SERS signal.
- N_{ref}: Number of molecules contributing to the reference signal.

Table S1. Estimated SERS enhancement factor (EF) values for different SERS substrate fabricated in this work

	SERS EFs (10^4) CV (1620 cm^{-1})	SERS EFs (10^4) R6G (1510 cm^{-1})	SERS EFs (10^4) DNTB (1340 cm^{-1})
**Substrate**	Au	Au	Au
**Au-P1**	0.48	0.25	0.14
**Au-P2**	0.99	0.54	0.2
**Au-P3**	1.58	0.89	0.53
**Au-P4**	2.99	1.42	0.82

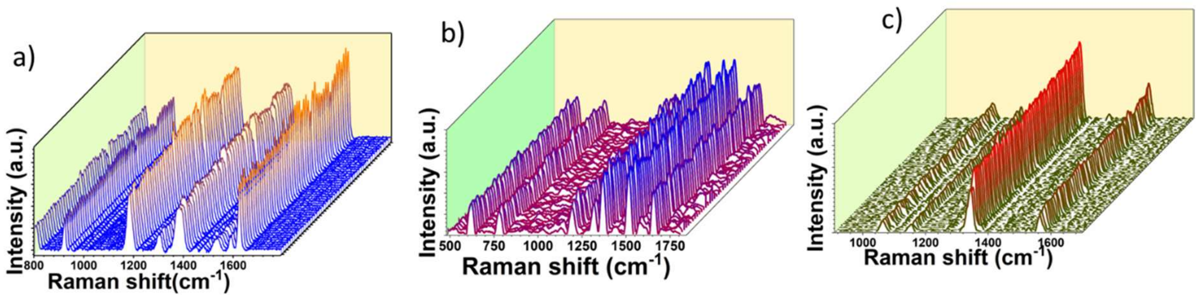

Figure S6. a-c) SERS spectra obtained of a) CV, b) R6G, and c) DTNB over 50-different spots in the 3D dendritic Au- substrate.

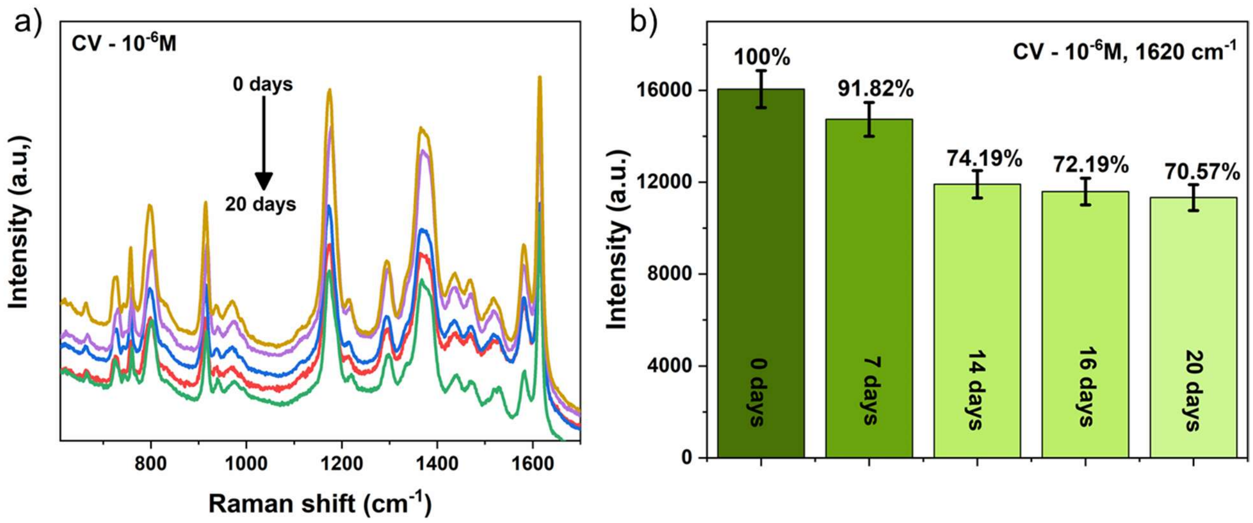

Figure S7. a) SERS spectra of CV molecules deposited 3D Au -SERS substrate over 20-days period. b) corresponding variation in the SERS peak intensity of CV (1620 cm^{-1}) over 20-days period.

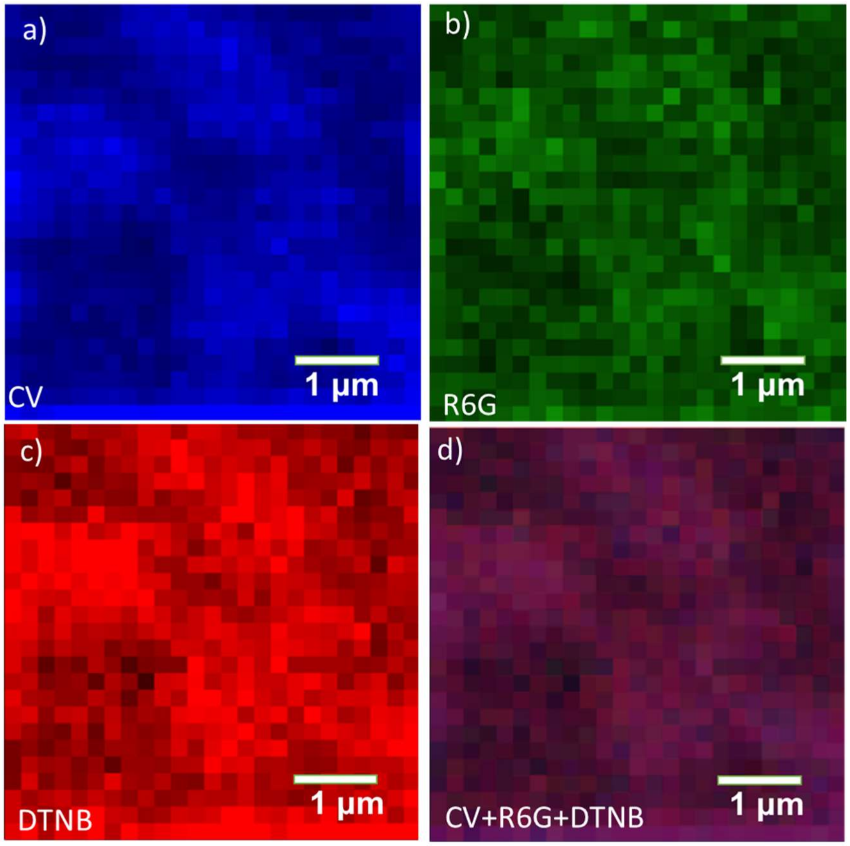

Figure S8: SERS-mapping image of multiplex imaging of three analyte molecules. a) CV, b) R6G, and c) DTNB, and d) overlapped image of CV+R6G+DTNB, respectively.

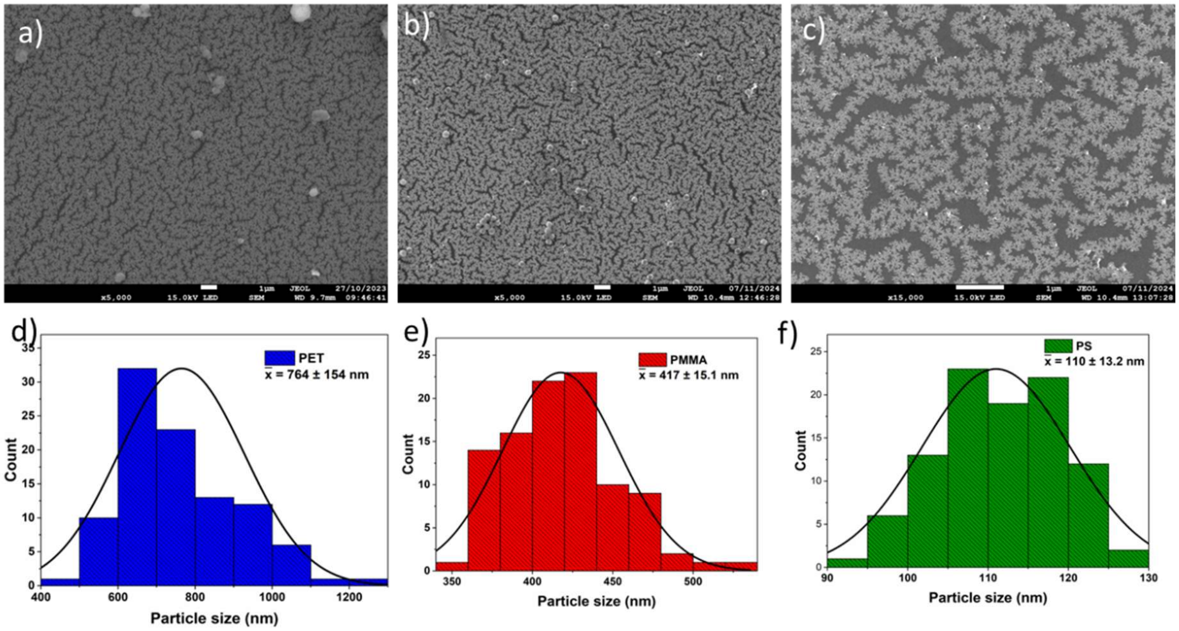

Figure S9. a-c) SEM images of the PET, PMMA, and PS nanospheres over the 3D dendritic Au SERS substrate, d-f) corresponding particle size distribution histograms of PET, PMMA and PS nanoplastics.

Table S2. Comparison of the SERS-detection parameters for Nanoplastics detection.

SERS substrate	Type of nanoplastics	Sensitivity	Size	References
Ag/ZnO@PDMS	Polystyrene	25 µg/mL	800 nm	Zhau et al.,[2]
Au NPs	Polystyrene	6.5 µg/mL	1-4 µm	Mikac et al.,[3]
Au pyramidal cavities	Polystyrene<br>PMMA	26.3 µg/mL<br>26.3 µg/mL	360 nm<br>500 nm	Xu et al.,[4]
Au NPs decorated sponge	4-mercaptopyridine	50 µg/mL	39-155 µm	Yin et al.[5]
AgNPs−$MgSO_4$	Polystyrene	100, 100 µg/mL	50 nm, 1µm	Zhou et al.,[6]
AuNSs@Ag@AAO	Polystyrene	50 µg/mL	400 nm	Le tal.,[7]
Flat Au nanoparticles	Polystyrene	0.03 µg/mL	30-1000 nm	Xing et al.[8]
3D Au film	PET,<br>PMMA<br>PS	$1.3x10^{-7} g/mL$<br>$4.5x10^{-6}$ g/mL<br>$6.8x10^{-7}$ g/mL	764 nm<br>410 nm<br>110 nm	this work<br>this work<br>this work

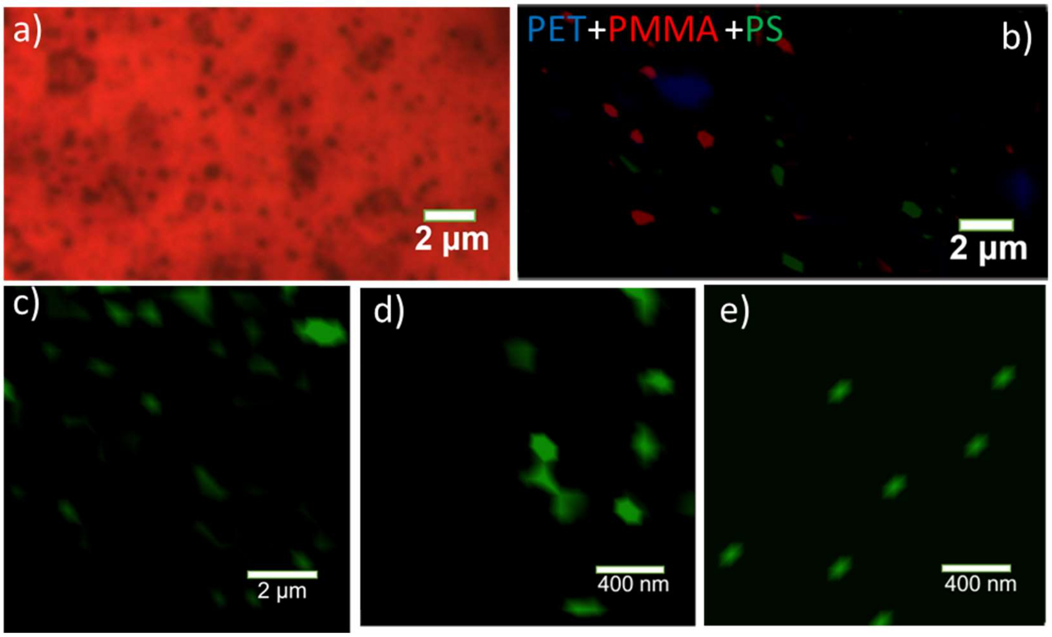

Figure S10. a-e) Multiplex SEM imaging of three different types of nanoplastics such as PET, PS, and PMMA with different sizes.

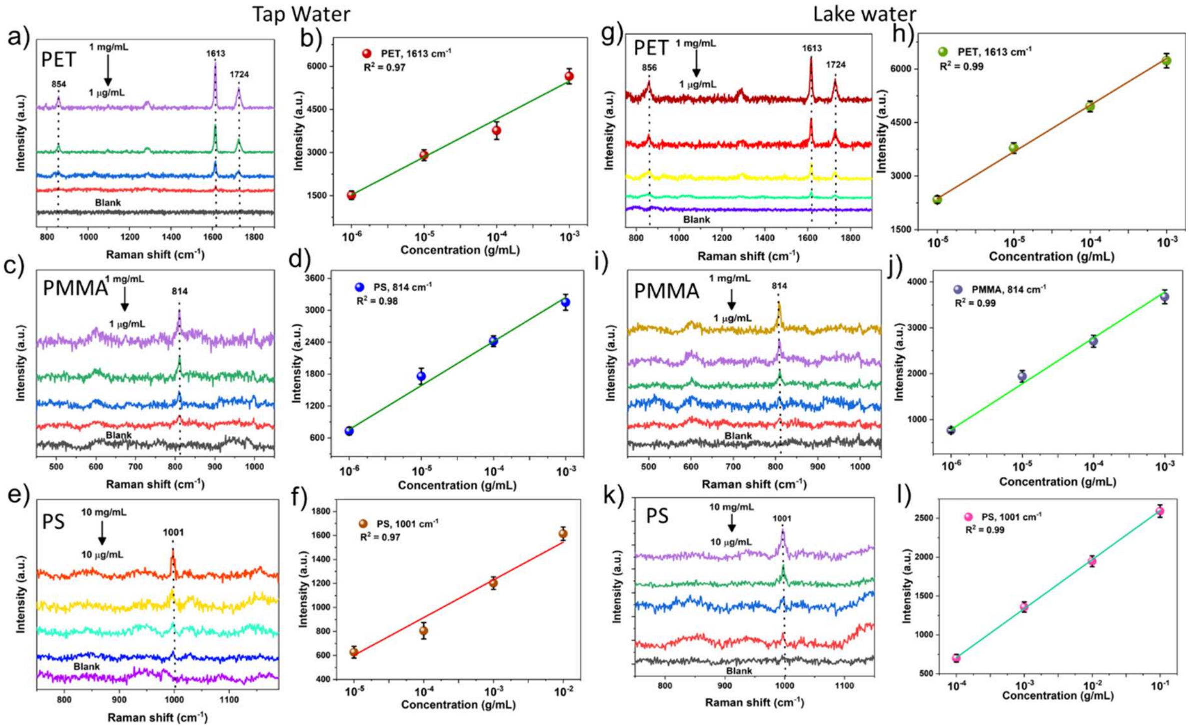

Figure S11. a-l) SERS spectra and corresponding linear plot of PET, PMMA, and PS nanoplastic particles with varied concentration in a-g) tap water and g-l) lake water, respectively.

Table S3. SERS sensitivity and LOD values of 3D Au substrate for detection of PET, PMMA and PS nanoplastics in real samples such as Tap water and lake water.

Tap water	
PET	$2.3x10^{-7} g/mL$
PMMA	$7.8x10^{-12} g/mL$
PS	$4.1x10^{-12} g/mL$
**Lake water**	
PET	$4.9x10^{-12} g/mL$
PMMA	$8.6x10^{-12} g/mL$
PS	$6.6x10^{-12} g/mL$

References:

(1) Liu, K.; Bai, Y.; Zhang, L.; Yang, Z.; Fan, Q.; Zheng, H.; Yin, Y.; Gao, C. Porous Au-Ag Nanospheres with High-Density and Highly Accessible Hotspots for SERS Analysis. *Nano Lett* **2016**, *16*, 3675–3681.

(2) Zhu, Z.; Han, K.; Feng, Y.; Li, Z.; Zhang, A.; Wang, T.; Zhang, M.; Zhang, W. Biomimetic Ag/ZnO@PDMS Hybrid Nanorod Array-Mediated Photo-Induced Enhanced Raman Spectroscopy Sensor for Quantitative and Visualized Analysis of Microplastics. *ACS Appl Mater Interfaces* **2023**, *15* , 36988–36998.

(3) Mikac, L.; Rigó, I.; Himics, L.; Tolić, A.; Ivanda, M.; Veres, M. Surface-Enhanced Raman Spectroscopy for the Detection of Microplastics. *Appl Surf Sci* **2023**, *608*, 155239.

(4) Xu, G.; Cheng, H.; Jones, R.; Feng, Y.; Gong, K.; Li, K.; Fang, X.; Tahir, M. A.; Valev, V. K.; Zhang, L. Surface-Enhanced Raman Spectroscopy Facilitates the Detection of Microplastics <1 Mm in the Environment. *Environ Sci Technol* **2020**, *54* , 15594–15603.

(5) Yin, R.; Ge, H.; Chen, H.; Du, J.; Sun, Z.; Tan, H.; Wang, S. Sensitive and Rapid Detection of Trace Microplastics Concentrated through Au-Nanoparticle-Decorated Sponge on the Basis of Surface-Enhanced Raman Spectroscopy. *Environmental Advances* **2021**, *5*, 100096.

(6) Zhou, X. X.; Liu, R.; Hao, L. T.; Liu, J. F. Identification of Polystyrene Nanoplastics Using Surface Enhanced Raman Spectroscopy. *Talanta* **2021**, *221*, 121552.

(7) Lê, Q. T.; Ly, N. H.; Kim, M. K.; Lim, S. H.; Son, S. J.; Zoh, K. D.; Joo, S. W. Nanostructured Raman Substrates for the Sensitive Detection of Submicrometer-Sized Plastic Pollutants in Water. *J Hazard Mater* **2021**, *402*,123499

(8) Xing, F.; Duan, W.; Tang, J.; Zhou, Y.; Guo, Z.; Zhang, H.; Xiong, J.; Fan, M. Superhydrophobic Surface-Enhanced Raman Spectroscopy (SERS) Substrates for Sensitive Detection of Trace Nanoplastics in Water. *Anal. Chem.* 2025, 97,2293–2299
